# Tumor-derived extracellular vesicles modulate innate immune responses to affect tumor progression

**DOI:** 10.3389/fimmu.2022.1045624

**Published:** 2022-11-02

**Authors:** Siqi Wang, Jiaxin Sun, Raha M. Dastgheyb, Zhigang Li

**Affiliations:** ^1^ Scientific Research Centre, The Seventh Affiliated Hospital, Sun Yat-sen University, Shenzhen, China; ^2^ School of Medicine, Sun Yat-sen University, Shenzhen, China; ^3^ School of Medicine, Johns Hopkins University, Baltimore, MD, United States

**Keywords:** macrophages, dendritic cells, neutrophils, natural killer cells, tumor-derived extracellular vesicles (TDEVs), tumor progression

## Abstract

Immune cells are capable of influencing tumor progression in the tumor microenvironment (TME). Meanwhile, one mechanism by which tumor modulate immune cells function is through extracellular vesicles (EVs), which are cell-derived extracellular membrane vesicles. EVs can act as mediators of intercellular communication and can deliver nucleic acids, proteins, lipids, and other signaling molecules between cells. In recent years, studies have found that EVs play a crucial role in the communication between tumor cells and immune cells. Innate immunity is the first-line response of the immune system against tumor progression. Therefore, tumor cell-derived EVs (TDEVs) which modulate the functional change of innate immune cells serve important functions in the context of tumor progression. Emerging evidence has shown that TDEVs dually enhance or suppress innate immunity through various pathways. This review aims to summarize the influence of TDEVs on macrophages, dendritic cells, neutrophils, and natural killer cells. We also summarize their further effects on the progression of tumors, which may provide new ideas for developing novel tumor therapies targeting EVs.

## 1 Introduction

Extracellular vesicles (EVs) usually refer to a group of heterogeneous, cell-released, double-membraned 150-1000 nm structure vesicles (1, 2). To address the considerable discrepancy in the definitions of EV subtypes, the International Society for Extracellular Vesicles (ISEV) suggested using “small EVs” (sEVs) for EVs under 200nm and “medium/large EVs” (m/lEVs) for EVs larger than 200nm in the position paper published in 2018 (1). In this article, the term “EVs” represents all cell-derived extracellular membrane vesicles (though most studies investigate sEVs).

The biogenesis of EVs has been well characterized (3). It is known that EVs originate from endosomes that are divided into 3 parts: early endosomes, late endosomes, and recycling endosomes. Invaginated vesicles first form intraluminal vesicles and integrate their cargoes into early endosomes. This cargo can consist of proteins, nucleic acids, and lipids, etc. Endosomes that internalize cargo to be recycled are classified as “recycling endosomes”. The rest of the early endosomes are subsequently transformed into late endosomes (4), also called multi-vesicular bodies (MVBs) (5), while dividing their cargo into vesicles budding in the lumen of the late endosomes. Four multi-protein complexes, endosomal sorting complexes required for transport (ESCRT) 0, I, II, and III, are involved in this step (6–8). ESCRT-0 has an association with cargo gathering in a ubiquitin-dependent process. The recruitment of ESCRT-I and II promotes endosomal membrane budding and ESCRT III is essential for completing budding. If the cargoes are destined to be degraded, the late endosomes will fuse with lysosomes and their cargoes will be digested. Other late endosomes carrying contents that are fated to be exported fuse with the plasma membrane and their internal vesicles are exported as EVs. Some ESCRT-independent mechanisms of EV biogenesis have also been reported, such as the biogenesis mechanism related to ceramide signaling pathways (9, 10).

Loaded with specific proteins, nucleic acids, and lipids, EVs play an important role in intercellular communication. In the 1990s, Raposo et al. reported that highly enriched MHC II molecules from Epstein-Barre Virus (EBV)-transformed B cell-derived EVs provoked certain CD4+ T cell clones, demonstrating EVs to be the critical mediator of intercellular communication (11). The binding specificity of EVs and recipient cells may be endowed by ligands expressed on their membranes. Thus, EVs can be used as potential targets of biomarker testing and transporters of drugs and genes.

Numerous recent studies have found that EVs are closely related to cancer progression (12–14). Tumor-derived EVs (TDEVs) take part in the formation of the tumor microenvironment (TME), epithelial-mesenchymal transition (EMT), angiogenesis, and metastasis. EMT and angiogenesis are both crucial processes for tumor metastasis and are regulated by EVs. For example, by interacting with endothelial cells TDEVs display a capacity for promoting angiogenesis by transporting activated epidermal growth factor receptor (EGFR) (15). Moreover, EVs facilitate tumor metastasis by mediating extracellular matrix (ECM) remodeling. Matrix metalloproteinase (MMP) is the major performing protein in tissue remodeling (16) and EVs derived from glioblastoma tumors have been found to increase the expression of MMP14 RNA in microglia (17). It has been found that macrophage migration inhibitory factor (MIF) existing in EVs derived from pancreatic ductal adenocarcinoma (PDAC) formed a pre-metastatic niche in the liver thus advancing liver metastasis (18). In addition, TDEVs can deliver their cargo to cancer cells, immune cells, and stromal cells. TDEVs exert a dual effect on tumor development by inhibiting natural killer (NK) cytotoxicity, mediating neutrophil differentiation, and influencing dendritic cell function. In many cases, TDEVs stimulate the pro-inflammatory M2 phenotype differentiation of macrophages and create an immunosuppressive microenvironment in tumor tissue. PD-L1 packed in TDEVs prevents T cell activation and stops tumor cells from being identified and killed (19, 20). These studies all provide evidence that TDEVs are engaged in many processes of tumor progression that affect immune cell functions.

Innate immune cells can be divided into classical innate immune cells, innate lymphoid cells (ILCs), and innate-like lymphocytes (ILLs). Classical innate immune cells include monocytes, macrophages, conventional dendritic cells, granulocytes, and mast cells. Here we review the studies focusing on the modulatory functions of TDEVs on four kinds of innate immune cells: macrophages, dendritic cells, neutrophils of granulocytes, and natural killer (NK) of ILCs.

Macrophages are differentiated from monocytes that migrate to tissues and organs under the influence of chemokines such as monocyte chemoattractant protein 1 (MCP-1). Induced by pathogens or different types of cytokines in the local microenvironment, monocytes differentiate into two macrophage subsets with different functional properties: type-1 macrophage (M1, classically activated macrophage) and type-2 macrophage (M2, alternatively activated macrophage). Compared with M1, M2 commonly accounts for a larger proportion of TME in solid tumors and results in tumor immune escape (21). Macrophages perform a variety of important functions including: phagocytosis and sterilization, inflammatory reaction, antigen-presenting, and immune regulation. Thus, macrophages are fundamental for the development and progression of tumors.

Dendritic cells (DCs) are functionally sorted as conventional DCs (cDCs), plasmacytoid DCs (pDCs), and monocyte-derived inflammatory DCs (moDCs) while the last type only appears during inflammation (22). cDCs generally participate in immunity as antigen-presenting cells (APC), acting as a bridge between adaptive and innate immune systems. They are essential for the induction and maintenance of anti-tumor immunity. In TME the antigen-presenting function is impaired (23). Depending on distinct environmental signals, tumor-infiltrating dendritic cells (TIDC) display either anti-tumor or pro-tumor functions. In most cases, TIDCs exhibit a tolerogenic phenotype under the impact of immune-suppressive factors like vascular endothelial growth factor (VEGF), IL10, TGFβ, and prostaglandin E2 (PGE2), subduing Th1-activating ability while enhancing Th2 and Treg responding (24). As a special lineage of DCs, pDCs are poor in antigen presentation and strong in IFN production. They contribute to tumor growth probably by activating Treg and forming an immune-subversive environment (25).

Neutrophils account for 70-80% of peripheral granulocytes. They possess a high generation rate of 1×10^7^ per minute but are short-lived (about 2-3 days in circulation). Within the cancer framework, neutrophils show N1 and N2 phenotypes, respectively acting as tumor suppressors and tumor promoters (22). Neutrophils are pro-inflammatory in the early stages of the tumor, but gradually display the immunosuppressive phenotype as the tumor progresses (26). They produce reactive oxygen/nitrogen species (ROS/RNS) to regulate inflammation, secret neutrophil elastase (NE) and MMP8/9 to accelerate invasion, release Oncostatin-M to encourage angiogenesis, and make PGE2 to promote tumor development (27).

NK cells are a subset of ILCs. There is a plethora of evidence indicating that NK receptor NKG2D assists the immune system in recognizing tumors. NK cells kill target cells and restrain primary tumor progression through various pathways including: antibody-dependent cell-mediated cytotoxicity (ADCC), Fas/FasL pathway, perforin/granzyme pathway, and release of cytokines such as TNF (28). Nevertheless, the killing efficiency of NK is limited due to the presence of TGF-β in plasma of both solid and hematological tumors (29–31). TGF-β can reduce the expression of activating receptors (including NKG2D, NKp30 and NKp46) and upregulate the expression of the inhibitory receptor NKG2A (29–31).

In the following sections we discuss the diverse impacts of TDEVs on innate immune cells, which including macrophages, dendritic cells, neutrophils, and NK cells. We further summarize the effects of these regulated immune cells on tumor progression. We will focus on the cargoes of TDEVs which modulate innate immunity is the promising novel targets for tumor diagnosis and treatment.

## 2 Macrophage

Macrophages have multiple functions, including phagocytosis and sterilization, and participation in inflammatory reactions, presenting antigens, and immune regulation (32). They recognize antigenic foreign bodies through surface pattern recognition receptors (PRR) and opsonic receptors, swallowing pathogens into the cell through receptor-mediated endocytosis. Sterilization is conducted by reactive oxygen intermediate (ROI) and reactive nitrogen intermediate (RNI), or directly by lysosomes and accumulated lactic acid. IgG Fc receptors on the surface of activated macrophages mediate ADCC to kill tumor cells or virus-infected cells (33). Activated macrophages also synthesize and secret chemokines and cytokines to participate in the inflammatory response. M1 and M2 macrophages have distinct secretion profiles (34). M1 macrophages tend to release chemokines (such as CCL2, CCL3, and CXCL8) and pro-inflammatory cytokines (such as IL-6, TNF-α, and IL-1β (35–37), while M2 macrophages produce anti-inflammatory factors like IL-10 and TGF-β more (34, 38). Peripheral organ-resident macrophages swallow pathogens, process them into small immunogenic peptides, present peptide fragments to the cell surface for recognition by CD4+ Th cells and trigger adaptive immune response (39, 40). Macrophages play a dual role in tumor immunity (41). On the one hand, macrophages induce specific anti-tumor responses by presenting antigens as professional APCs. Activated macrophages kill tumor cells through non-specific phagocytosis or ADCC, and also indirectly by secreting TNF, NO, and other cytotoxic factors. On the other hand, macrophages are polarized into TAMs under the influence of certain molecules released by tumor cells, promoting tumor development.

### 2.1 TDEVs regulate the polarization of macrophage

It is well known that macrophages participate in innate immune responses (42). CD14 and CD68 are the characteristic surface markers of the human macrophages (43–46). Circulating monocytes become macrophages when infiltrating into tissues. Based on the expression of specific surface markers, monocyte-derived macrophages can be polarized and divided into two phenotypes including M1 and M2 (47). In humans, M1 macrophages specifically express CD64, CD86, MARCO, CXCL9, CXCL10, CXCL11, NOS2, and SOCS1 (46, 48–51) on the surface, while M2 macrophages are identified by expressing TGM2, CD23, CD163, CD206, ARG1 and CCL22 (44, 46, 48, 49, 52–54). M1 macrophages, also known as classically activated macrophages, are induced by Th1 cytokines such as IFN-γ, IL-1β, and LPS. They secrete pro-inflammatory cytokines and function as anti-tumor cells. M1 macrophages bear the antigen-presenting ability so that they can activate adaptive immunity and bring about tissue damage (39, 40, 42, 55, 56). Secreting inhibitory factors like IL-4, IL-10, and IL-13, M2 macrophages show pro-tumor activity by promoting local immunosuppression, angiogenesis, and metastasis (57, 58). Tumor-associated macrophages (TAM), taking up 50% of the host infiltrating cells in TME, are usually considered as M2 phenotype (59). Meanwhile, there are many subsets in between that have yet to be clarified, expressing both M1 and M2 markers (60–62). Studies have shown that the polarization of macrophages in TME towards M1 or M2 can be promoted by contents in TDEVs. In **Table 1** we summarize previous studies that investigate TDEVs cargoes in regulating the polarization of the macrophage, including miRNAs, lncRNAs, circRNAs, and proteins.

**Table 1:** Cargoes of TDEVs in promoting the polarization of macrophages.

Cargo		Cancer type	Mechanism	Polarization	References
miRNAs	miR-21	Bladder cancer	PI3K/AKT pathway	M2	(63)
	miR-25-3p, miR-130b-3p, miR-425-5p	CRC	PTEN/PI3K/AKT pathway	M2	(64)
	miR-301a-3p	Esophageal squamous cancer	PTEN/PI3K/AKT pathway	M2	(65)
	miR-222	Adriamycin-resistant breast cancer	PTEN/PI3K/AKT pathway	M2	(66)
	miR-19b-3p	Lung adenocarcinoma	Hippo pathway	M2	(67)
	miR-423-3p	Cervical cancer	Blocking the expression of CDK4 mRNA	M2	(68)
	miR-21	Hypoxic tumor cells, HNSCC, bladder cancer	–	M2	(63, 69, 70)
	miR-138-5p	Breast cancer	Inhibiting KDM6B expression	M2	(71)
	miR-770	NCSLC	Targeting MAP3K1	M1	(72)
	miR-130	Breast cancer	M2 macrophages reprogramming	M1	(73)
	miR-9	HPV + HNSCC	–	M1	(74)
lncRNAs	PCAT6	NSCLC	–	M2	(75)
	ARSR	Renal cell carcinoma	STST3 pathway	M2	(76)
	HMMR-AS1	HCC	MiR-147a/ARID3A axis	M2	(77)
	TP73-AS1	Nasopharyngeal carcinoma	Binding with miR-342-3p	M2	(78)
	FGD5-AS1	Pancreatic cancer	STAT3/NF-κB pathway	M2	(79)
	ELFN1-AS1	Osteosarcoma	Sponging miR-138-5p and miR-1291	M2	(80)
	HCG18	Gastric cancer	Sponging miR-875-3p	M2	(81)
circRNAs	hsa-circ-0048117	Esophageal squamous cancer	Sponging miR-140	M2	(82)
	hsa_circ_0017252	Gastric cancer	Sponging miR-17-5p	M2	(83)
	circFARSA	NSCLC	PTEN/PI3K/AKT pathway	M2	(84)
	circ_0001142	Breast cancer	Circ_0001142/miR-361-3p/PIK3CB pathway	M2	(85)
	circPVT1	Lung cancer	MiR-124-3p/EZH2 axis	M2	(86)
	circSAFB2	Renal cell carcinoma	MiR-620/JAK1/STAT3 axis	M2	(87)
	circNEIL3	Glioma	Stabilizing IGF2BP3	M2	(88)
protein	CSF-1, MCP-1/CCL2, EMAP2/AIMP1 and LTA4H	Melanoma, skin squamous cell carcinoma and lung cancer	–	M2	(89)
	gp130	Diffuse large B-cell lymphoma	STAT3 pathway	M2	(90)
	leptin	Gallbladder cancer	STAT3 pathway	M2	(91)
	PTPRO	Breast cancer	STAT3 pathway	M1	(92)
	RNF126	Nasopharyngeal carcinoma	PTEN/PI3K/AKT pathway	M2	(93)
	ANLN	HNSCC	PTEN/PI3K/AKT pathway	M2	(94)
	TIM-3	Osteosarcoma	Increases the expression of N-cadherin and Vimentin, decreases E-cadherin expression	M2	(95)
		Melanoma	–	M2	(96)
	PD-L1	Oral squamous carcinoma cells	Up-regulate the expression of PD-L1	M2	(97)
	ICAM-1	PDAC	–	M2	(98)
	CXCL14	Prostate cancer	NF-κB pathway	M2	(99, 100)
	αvβ6	Prostate cancer	–	M2	(101)
	αvβ6 negative	Prostate cancer	–	Prevent M2 polarization	(101)

#### 2.1.1 miRNAs cargo in TDEVs

Tumor-derived miRNAs assist in M2 polarization, thereby enhancing tumor proliferation through intercellular dialogue. The PTEN/PI3K/AKT signaling pathway is a common mechanism by which miRNAs regulate macrophage polarization. In macrophages, activating the PI3K/AKT pathway leads to the activation of signal transducer and activator of transcription 3 (STAT3), which is crucial for the differentiation of macrophages into M2 phenotype (102, 103). For example, miR−21 is carried in human bladder T24 cancer cell-derived EVs and promotes macrophages to M2 polarization, which through inhibited PI3K/AKT dephosphorylation leads to increase STAT3 activation (63). The miRNAs (miR-25-3p, miR-130b-3p, miR-425-5p) transferred from colorectal cancer (CRC) to macrophages *via* EVs can enhance M2 polarization by adjusting PTEN through the activation of PI3K/Akt signaling pathway, which finally leads to tumor EMT, angiogenesis, and liver metastasis (64). The PTEN/PI3K/AKT signaling pathway is also the target of miR-301a-3p from esophageal squamous cancer cell-derived EVs (65) and miR-222 from Adriamycin-resistant MCF-7 breast cancer cell-derived EVs (66) to induce M2 polarization. Chen et al. have reported that highly enriched miR-19b-3p in lung adenocarcinoma-derived EVs promoted M2 polarization through the Hippo pathway (67). MiR-19b-3p targets PTPRD, suppresses the PTPRD-mediated dephosphorylation of STAT3, activates STAT3, and induces polarization (67). In addition, Yan et al. have found that cervical cancer cell-secreted EVs transported miR-423-3p to stimulate macrophage M2 polarization by blocking the expression of CDK4 mRNA (68).

EVs produced by hypoxic tumor cells are enclosed with miR-21 and promote the transformation from monocyte to M2-polarized macrophage, and form an immunosuppression environment in TME (69). The same effect of EV miR-21 has also been observed in head and neck squamous cell carcinoma (HNSCC) and bladder cancer (63, 70). Moreover, breast cancer cell-derived EVs carrying miR-138-5p suppress M1 polarization and upgrade M2 polarization through the inhibition of epigenetic factor lysine demethylase 6B (KDM6B) expression in a suspension coculture system comprising breast cancer cells and macrophages (71).

Notably, some miRNAs in EVs are capable of suppressing tumor progression. Non-small cell lung carcinoma (NSCLC) cell-derived extracellular vesicular miR-770 has been confirmed to inhibit M2 macrophage polarization by targeting MAP3K1, which in turn prevents tumor invasion (72). MiR-130 originating from breast cancer cells leads to a reprogramming from M2 macrophages to M1 macrophages while the upregulation of M1 specific markers and downregulation of M2 specific markers were tested. After reprogramming, the phagocytic function of macrophages is enhanced and the ability to metastasize is impaired (73). Furthermore, miR-9-enriched EVs derived from HPV + HNSCC promote M1 phenotype polarization and then improve the radiosensitivity of HNSCC (74).

#### 2.1.2 lncRNAs cargo in TDEVs

lncRNAs are RNAs longer than 200 nucleotides with a relatively restricted protein-coding capacity (104). lncRNAs can promote M2 polarization in many ways. Chen et al. found that the knockdown of lncRNA PCAT6 could prevent its positive effect on M2 polarization and in turn accelerated NSCLC development (75). lncARSR-containing EVs derived from renal cell carcinoma achieve a similar effect *via* the STAT3 pathway (76). MiR-147a/ARID3A axis is activated under hypoxia condition by hepatocellular carcinoma (HCC)-derived EVs delivering lncRNA HMMR-AS1 (77). Mechanistically, HMMR-AS1 interacts with miR-147a to reduce ARID3A degradation after which M2 macrophage polarization and the development of HCC are promoted (77). In addition, lncRNA TP73-AS1 is highly expressed in nasopharyngeal carcinoma cell-derived EVs, which binds to miR-342-3p, promotes the M2 polarization of macrophages, and reinforces the motility and microtubule formation of macrophages (78). Furthermore, FGD5-AS1 enriched in pancreatic cancer cell-derived EVs mediates M2 polarization of macrophages by activating STAT3/NF-κB pathway (79).

Interestingly, previous studies have found that lncRNAs can bind with miRNA to remove them from circulation and then promote tumor growth. For example, lncRNA ELFN1-AS1 exists in osteosarcoma cell-derived EVs sponge miR-138-5p and miR-1291 to suppress the M2 polarization (80). The down-regulation of miR-875-3p reached by lncRNA HCG18 in gastric cancer cell-derived EVs facilitates the M2 polarization of macrophages (81).

In addition to regulating macrophage polarization, lncRNAs also cause chemoresistance. It has been confirmed that glioblastoma cell-derived extracellular vesicular lnc-TALC induces temozolomide resistance by binding to ENO1 and phosphorylating p38 MAPK (105). LncRNA PART1 in esophageal squamous cell carcinoma cell-derived EVs competitively combines with miR-129 to raise the expression of Bcl-2 in esophageal squamous cell carcinoma cells, thus inducing the drug resistance of tumor cells to gefitinib (106). This indicates that lncRNA in EVs may serve as a promising therapeutic target for cancer patients.

#### 2.1.3 circRNAs cargo in TDEVs

Emerging studies indicate that circRNAs play a critical role in tumor-induced immune responses. CircRNAs sometimes serve as miRNA sponges, binding with them and removing them from circulation. For instance, esophageal squamous cancer cell-derived hsa-circ-0048117 is transmitted to macrophages, works as a miR-140 sponge, and mediates M2 polarization (82). Hsa_circ_0017252-containing EVs of gastric cancer inhibits M2 polarization and tumor development through sponging miR-17-5p (83).

Various signaling pathways are involved in the regulation of macrophage polarization. Tumor-derived circFARSA delivered by EVs regulates M2 polarization *via* PTEN/PI3K/AKT pathway to raise the metastatic potential of NSCLC (84). Circ_0001142 carried by breast cancer cell-released EVs influences macrophages’ autophagy and polarization *via* circ_0001142/miR-361-3p/PIK3CB pathway (85). Circ_0001142, targeting PIK3CB, is capable of activating the PI3K/AKT - an effect that can be reversed by miR-361-3p (85). There are other signaling pathways circRNAs interfere with, resulting in M2 polarization and tumor progression, such as the miR-124-3p/EZH2 axis targeted by circPVT1 in EVs and miR-620/JAK1/STAT3 axis targeted by circSAFB2 in EVs. Lung cancer cell-derived circPVT1 increases EZH2 expression by downregulating miR-124-3p expression so that macrophages are induced to polarize towards an M2-like phenotype (86). CircSAFB2 in renal cell carcinoma cell-derived EVs functioned as a miR-620 sponge while JAK1 and STAT3 protein levels were tested markedly lower after co-culturing with miR-620 mimics (87). This correlates with the result that renal cell carcinoma cell-derived EVs induce macrophages express a higher level of JAK1 and STAT3 protein, and miR-620 can prevent the JAK1 and STAT3 expression (87). In addition, circNEIL3 has been shown to contribute to tumor progression by stabilizing the oncogenic protein IGF2BP3, which is packaged by hnRNPA2B1 in glioma cells and transported to TAMs (88).

#### 2.1.4 Protein cargo in TDEVs

Previous studies have found that proteins carried by TDEVs affect the polarization of macrophages in multiple ways. Park et al. reported a 3-4-fold increase of total EV-containing protein per cell under hypoxia conditions in melanoma cell lines (B16-F0 and A375), skin squamous cell carcinoma cell line (A431), and lung cancer cell line (A549). Several abundant proteins such as CSF-1, MCP-1/CCL2, EMAP2/AIMP1, and LTA4H were detected in these EVs, which help in monocyte/macrophage recruitment and M2 polarization (89). Diffuse large B-cell lymphoma-derived EVs are enriched in gp130, which functions as the activator of the STAT3 signaling pathway to stimulate downstream targets like BCL2, SURVIVIN, and BAX to promote M2 polarization (90). Similarly, leptin existing in the EVs derived from gallbladder cancer boosts M2 macrophage polarization by activating the STAT3 signaling pathway as well (91). On the contrary, protein tyrosine phosphatase receptor type O (PTPRO) in EVs produced by breast cancer cells induces M1 polarization *via* inactivating the STAT signaling pathway and then inhibits tumor migration (92). In addition, nasopharyngeal carcinoma-derived EVs containing RNF126 induce the M2 polarization of macrophages and contribute to the invasion and metastasis of tumors. Yu et al. have demonstrated that RNF126 degrades PTEN and provokes the PI3K/AKT pathway to regulate macrophage polarization (93). Furthermore, HNSCC-derived EVs carrying Anillin, actin-binding protein (ANLN), induced M2 polarization of macrophages *via* PTEN/PI3K/AKT signaling pathway (94).

The T-cell immunoglobulin and mucin domain 3 (TIM-3), also known as HAVCR2, has been proved to express in activated Th1 cells, Tregs, macrophages, dendritic cells, NK cells, and tumor cells (107, 108). Cheng Z. et al. found that the TIM-3 in osteosarcoma cells-derived EVs promoted M2 polarization, tumor invasion, metastasis, and EMT (95). The underlying mechanism is that TIM-3 increases the expression of N-cadherin and Vimentin, but decreases that of E-cadherin in infiltrated monocytes (95). While Li et al. also demonstrated that TIM-3 enriched in melanoma cell-derived EVs facilitated M2 type differentiation but the mechanism remained elusive (96).

It is well known that the plasma membrane-associated receptors play an important role in the function of immune cells. The surface receptors can also be packed in TDEVs and exert an influence on macrophages in different manners. Yuan et al. revealed the mechanism underlying endoplasmic reticulum stress and tumor development. They found that endoplasmic reticulum stress led oral squamous cell carcinoma (OSCC) to produce EVs loaded with PD-L1 and up-regulate the expression of PD-L1 in macrophages, thus driving the M2 macrophage polarization (97). In addition, ICAM-1 enriched in PDAC-derived EVs binds to CD11c on the surface of macrophages. Besides inducing M2 phenotype differentiation, these EVs also up-regulate the secretion of pro-tumoral molecules like VEGF, MCP-1, IL-6, IL-1β, MMP-9, and TNFα in macrophages exposed (98). Moreover, evidence indicates that prostate cancer cell-derived EVs loading CXCL14 promotes M2 polarization through activating NF-κB signaling, which is a key regulator of macrophage function and tumor progression (99, 100). Prostate cancer cells release two kinds of EVs, including αvβ6 (a surface receptor of the integrin family) positive and negative expression EVs. The αvβ6-positive EVs promote the M2 type differentiation of peripheral blood mononuclear cells, while the negative ones prevent this effect (101).

### 2.2 TDEVs regulate the pro-inflammatory responses of macrophage

**Figure 1:**
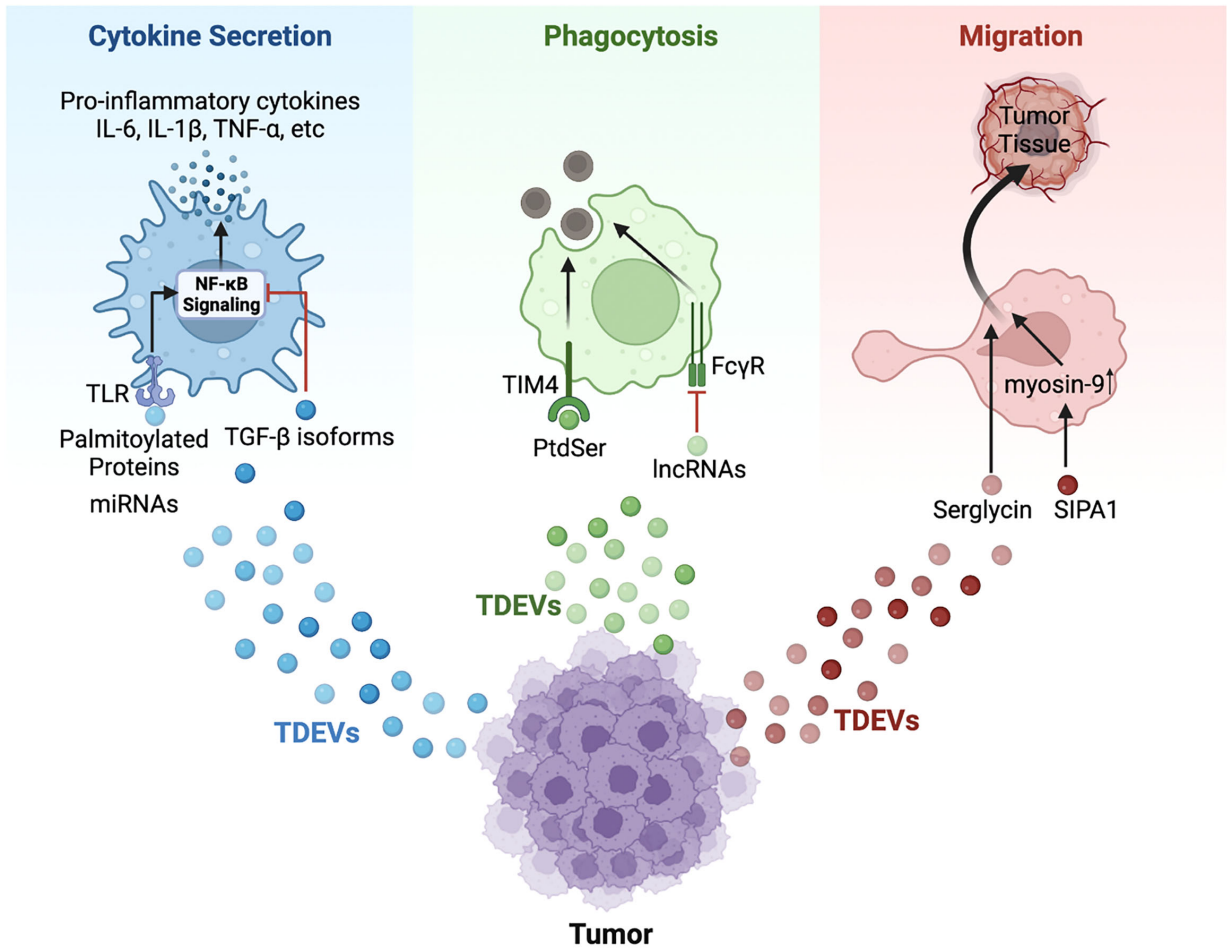
Schematic model of TDEVs in regulating the functions of macrophage. TDEVs can modulate the cytokine secretion, phagocytosis, and migration of macrophages *via* various signaling pathways. For cytokine secretion (Blue): Palmitoylated proteins carried by gastric cancer-derived EVs bind with TLR2 on the surface of macrophages to further activate NF-κB signaling, leading to an elevated level of pro-inflammatory cytokines including IL-6, IL-1β, TNF-α and so on. Similarly, miRNAs in EVs secreted by lung cancer and breast cancer bind with TLR7/8 to induce pro-inflammatory cytokines secretion *via* the NF-κB pathway. However, HNSCC-derived EVs cargo TGF-β isoform inhibits NF-κB signaling to down-regulate the expression of pro-inflammatory cytokines. For phagocytosis (Green): PtdSer in apoptotic tumor-derived EVs binds with receptors such as TIM-4 to promote the phagocytosis of apoptotic cells by macrophages. However, lncRNA TUC399 contained in HCC-derived EVs down-regulates the FcγR-mediated phagocytosis. For migration (Red): TDEVs from myeloma containing serglycin can augment macrophage migration, and SIPA1 in breast cancer-derived EVs induces elevated expression of myosin-9 in macrophages, which contributes to migration.

Macrophages can secret pro-inflammatory factors to regulate fibrosis, metabolism, cellular debris, and T cell function directly or indirectly (109). For example, IL-6 released from macrophages dominates in phosphorylating STAT3 and further promotes tumor growth and metastasis (110, 111). The level of pro-inflammatory cytokines can be up-regulated by TDEVs in gastric cancer, breast cancer, lung cancer, CRC, melanoma, and OSCC (112–119), leading to tumorigenesis and metastasis further. Inflammatory cytokines array showed that the expression of IL-6, CCL2, GCSF, and TNF-α was augmented by TDEVs, along with the phosphorylation of transcription factor NF-kB, which indicates that TDEVs stimulate the secretion of pro-inflammatory cytokines *via* NF-κB signaling pathway in gastric cancer, lung cancer, breast cancer and melanoma (112–115, 120). Various components of EVs exerting regulatory effects are being studied. For example, palmitoylated proteins on the surface of EVs are identified as a key factor in binding with macrophage surface protein Toll-like receptor (TLR) 2 to further phosphorylate NF-κB and activate inflammatory responses (113) (**Figure 1**). In addition, miRNAs also play an important role in modulating pro-inflammatory cytokines release. Lung cancer-derived EVs transporting miR-16, -21, -29a bind to TLR7/8 on the surface of macrophages to elicit phosphorylated activation of NF-κB, which induces an increase in transcription of pro-inflammatory cytokines including IL-6 (115). Similarly, miR-25-3p in breast cancer-derived EVs binding with TLR7/8 increases the expression of IL-6 and phosphorylated NF-κB (116). Moreover, oncogenic miR-183-5p in breast cancer-derived EVs are engulfed by macrophages and downregulate target gene PPP2CA expression by combining with the binding sequence which leads to a decrease in dephosphorylation of p65, consequently promoting IL-1β, IL-6, and TNF-α secretion (121).

The STAT3 pathway is engaged in the modulation by TDEVs. EVs released by endoplasmic reticulum-stressed liver cancer cells upregulate IL-6, IL-10, and MCP-1 levels but downregulate TNF-α levels in macrophages with an increase in p-JAK2 and p-STAT3 (119). Gp130 (IL-6 receptor) is carried by TDEVs and interacts with macrophages to induce the phosphorylation and translocation of STAT3 to the nucleus, leading to an elevated expression of IL-6 and a shape of the pro-tumor cancer environment in several human breast cancer cell lines (MDA-MB231, MDA-MB-468, Hs578T, and MCF7) (118).

### 2.3 TDEVs regulate the anti-inflammatory responses of macrophage

TDEVs present a double-sided sword: they participate in activation of inflammatory responses, but they can downregulate the expression of pro-inflammatory cytokines as well. HREV-positive EVs derived from two CRC cell lines (SK-CO1 and Caco-2) lead to a lower level of pro-inflammatory cytokine IL-1β and a higher level of anti-inflammatory cytokine IL-10 in the zebrafish model with a positive correlation between the concentration of HERV-positive EVs and anti-inflammatory responses (122). HNSCC-derived EVs down-regulate macrophage release of IL-1β, indicating that HNSCC-derived EVs block the activation of inflammatory responses. TGF-β isoforms composition is hypothesized to be the key factor *via* inhibiting the NF-κB signaling pathway (123) (**Figure 1**). Previous studies have also indicated that miRNAs play an important role in the modulation of cytokines. Li J. et al. found that EVs treated under hypoxia and released by lung cancer cells down-regulate the expression of pro-inflammatory cytokines IL-6 and IL-1A through cargo containing miR101 while targeting CDK8 and SUB1 (124). Moreover, lncRNAs act as the mediator of cytokines secretion through TDEVs. Li X. et al. found that HCC-derived EVs containing lncRNA TUC339 were engulfed by macrophage (THP-1 cell) and downregulate the secretion of IL-1β and TNF-α (125).

### 2.4 TDEVs regulate the macrophages phagocytotic function

Macrophages, as phagocytes, engulf apoptotic cells and debris that trigger immune responses to exert an anti-tumor effect (109). Previous studies have indicated that the phagocytotic activity of macrophages can be downregulated by TDEVs. EVs from metastatic osteosarcoma (K7M3 and DLM8) reduce the phagocytic function of alveolar macrophages *via* promoting TGF-β2 secretion, while there is no significant change in phagocytosis of macrophages taking up non-metastatic osteosarcoma (K7 and Dunn) -derived EVs (126). Along these same lines research conducted by Li X. and colleagues showed that HCC-derived EVs enriched in lncRNA TUC339 led to decreased FcγR-mediated phagocytosis in macrophages were found in (125). Furthermore, Gregory C.D. et al. found that apoptotic TDEVs contained phosphatidylserine (PtdSer) to bind with proteins such as T cell immunoglobulin and mucin domain-4 (TIM-4), which had a positive relation to phagocytosis of apoptotic cells (127) (**Figure 1**). These studies shed light on the relationship between phagocytosis in macrophages and TDEVs, however, the specific mechanism is still unclear.

### 2.5 TDEVs modulate the macrophages migration

During cancer development, macrophages migrate out of circulation and into the tumor milieu, triggering inflammation and tumor metastasis (128). EVs released by PDAC elicit migration to the liver of bone marrow-derived cells including macrophages, which follows an elevated level of TGF-β released by Kupffer cells (18). Myeloma-derived EVs with serglycin engulfed by macrophages augment migration as compared with serglycin-null EVs (129). In addition, it is reported that EVs from breast cancer cells (MDA-MB-231) expressing high levels of signal-induced proliferation-associated 1 (SIPA1) promote the migration of macrophages to tumor tissue (130) (**Figure 1**). SIPA1 binds to the promoter of the target gene MYH9 to upregulate the transcription of MYH9, and the enrichment of myosin-9 in EVs contributes to macrophage migration (**Figure 1**).

## 3 Dendritic cells

Dendritic cells (DCs), derived from hematopoietic stem cells, are identified as a vital kind of innate immune cells. As APCs, DCs recognize and swallow pathogens, subsequently presenting to immune cells like T cells to activate immune responses, by corresponding receptors and co-stimulatory molecules on the surface (131). In addition, DCs also secrete cytokines and chemokines capable of modulating the microenvironment and tumor development. Various functions of DCs are regulated by TDEVs, and interestingly they are capable of causing both anti-tumor and pro-tumor effects under certain conditions. DCs are divided into three subsets, classical DC (cDC), plasmacytoid DC (pDC), and monocyte-derived DC (mo-DC) (132). The former two are derived from common dendritic cell progenitors (CDPs), and mo-DC are derived from monocytes. Mature DCs express a higher level of functional molecules including co-stimulatory molecules (CD40, CD80, CD86), MHC II, pro-inflammatory cytokines, and CCR7 comparing to immature DCs, *via* the stimulus with GM-CSF, IFN-γ, IL-4 and pathogens (133).

### 3.1 Anti-tumor effect of DCs on responses to TDEVs

**Figure 2:**
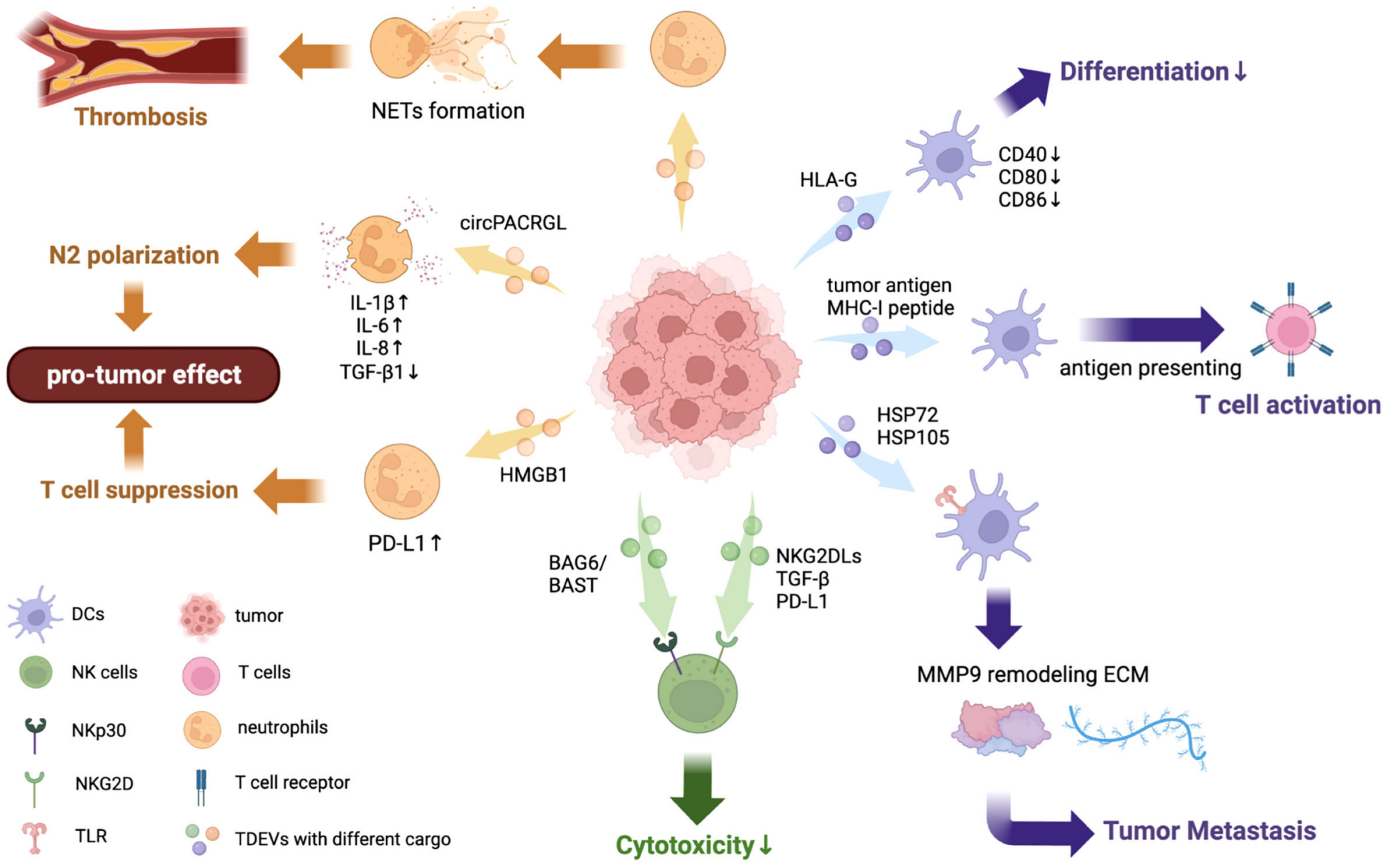
Effects of TDEVs in regulating DCs, neutrophils, and NK cell functions. TDEVs can interact with innate immune cells including DCs, neutrophils, and NK cells, exerting a dual effect in regulating their functions. TDEVs to DCs: EVs containing tumor antigen from HCC can transfer it to DCs and further activate T cell immunity, indicating its anti-tumor effect. TDEVs can impair DCs differentiation and maturation to suppress immune responses in some cancers, HLA-G is identified as a key factor in this negative regulation. Some TDEVs contain HSP72 and HSP105 bind with TLR on DCs to increase MMP9 expression, reorganizing ECM and contributing to tumor invasion. TDEVs to neutrophils: circRNA PACRGL in CRC-derived EVs are engulfed by neutrophils and down-regulate TGF-β1 expression, further inducing polarization to the N2 phenotype. TDEVs can also induce NET formation by neutrophils in a dose-dependent way, which further contributes to thrombosis. TDEVs containing HMGB1 can upregulate PD-L1 on neutrophils to suppress T cell immunity, exerting a pro-tumor effect. TDEVs to NK cells: BAG6/BAT3 on the surface of TDEVs can bind with NKp30 to suppress NK cytotoxicity *via* the nSmase2-dependent pathway. NKG2DLs, TGF-β, and other immunosuppressive proteins from TDEVs bind with NKG2Ds on the surface of NK cells to block NK cytotoxicity.

TDEVs augment anti-tumor activity by promoting the function of DCs in many cancers like melanoma, HCC, and colon carcinoma (120, 134, 135). It has been found that tumor antigen (TA) carried by EVs are a key factor in this process. HCC cell-derived EVs inhibit tumor growth by transferring HCC antigens and antigenic chaperones to DCs, which induces cytolysis and increases IFN-γ expression but decreases IL-10 and TGF-β expression. Furthermore, DCs treated with HCC cell-derived EVs activate T-cell immunity by presenting antigens (135). Similarly, EVs containing tumor antigen ErbB2 promotes the activation of CD8+T cell by DC (136). Melanoma cell-derived EVs carrying tumor-associated antigens (TAAs) promote DCs to express maturation marker CD86 and raise the anti-tumor activity in mouse models (137). In addition to whole-tumor antigens, MHC-I peptide complexes are also transferred to DCs by TDEVs in melanoma, resulting in the activation of cytotoxic T-lymphocytes (CTL) (138). The specific mechanism for this is still under investigation. The only known study to date is by Squadrito M.L. et al. who found that CRC-derived EVs internalized by DCs promoted the presentation of tumor antigens mediated by MHC-I, and extracellular vesicle-internalizing receptor (EVIR) played an important role in the binding and internalization of TDEVs by DCs (134) (**Figure 2**).

### 3.2 Immunosuppress effect of DCs on responses to TDEVs

A decrease in pro-inflammatory cytokine secretion but an increase in anti-inflammatory cytokines can be detected in DCs stimulated with TDEVs. In Lewis lung cancer (LLC) and breast cancer, surface markers including CD80, MHC-II, and CD86 are downregulated by TDEVs, indicating that DCs are immature. In addition, the cytokine expression in DCs is regulated by TDEVs as well. There is a decrease in TNF-α, IL-6, and IL-12, but an increase in Arginase I while the levels of IL-10 and IL-12p40 do not change significantly. In addition, chemokine receptor expression (which is essential for the migration of DCs) is inhibited by LLC cell-derived EVs (139). The expression of IL-6 is increased by TDEVs in breast cancer, lung cancer, and melanoma in various ways (140). HSP72 and HSP105 proteins on melanoma-derived EVs surface bind with TLR2 and TLR4 on DCs, causing the phosphorylation of ERK, JNK, p38, and NF-kB to induce expression of IL-6. IL-6 induces STAT phosphorylation to bind in the MMP9 promoter site and elevates the transcription of MMP9. Due to the function of reorganizing extracellular matrix by MMP9 (141), tumor cells invade other organs. PGE2 carried by prostate cancer-derived EVs binds to receptors EP2/EP4 on DCs to upregulate the CD73 expression, while the adenosine monophosphate (AMP)-depends on expressions of IL-12 and TNF-α decrease subsequently (142). However, the underlying mechanism still needs further investigation.

Notably, microRNAs in TDEVs can interfere with the function of DCs. MiR-212-3p carried by pancreatic cancer cell-derived EVs inhibited the expression of regulatory factor X-associated protein (RFXAP) to decrease MHC II on DCs and induce immune tolerance (143), while miR-203 induced the downregulation of TLR4 and cytokines in DCs such as TNF-α and IL-12 in pancreatic cancer (113). In head and neck cancer, TDEVs disrupt the maturation, viability, and migration of mono-CDs targeted by 133 miRNAs including miR-16, miR-23b, miR-24. CD80 and HLA-DR expression have been shown to exhibit a decrease in DCs after incubating with HNSCC cell-derived EVs (144) (**Figure 2**).

### 3.3 TDEVs modulate the differentiation of DCs

Wieckowski E. et al. found that antigen-processing machinery (APM) components including MIB1, IMP7, Tapasin, and Calreticulin were downregulated in monocytes after stimulation with TDEVs, indicating impaired differentiation from monocytes to DCs (145). Surface proteins that demonstrate the maturation of DCs decrease with the stimulation of TDEVs in melanoma, lung cancer, renal cancer, breast cancer, and thymoma (139, 146–149). Expressions of markers such as CD40, α5 integrin, CD80, CD86, and HLA-DR are downregulated in monocyte-derived DCs after co-incubation with renal cancer derived-EVs carrying HLA-G, which can be inhibited by anti-HLA-G-antibody. This confirmed the negative-regulatory role of EVs with HLA-G in DCs differentiation (146) (**Figure 2**). Hendrik Gassmann and colleagues proposed a model where Ewing sarcoma-derived EVs carrying RNA and protein activate myeloid cell pathology and induce the secretion of pro-inflammatory cytokines such as IL-6, IL-8, and TNF, which modulates the differentiation of myeloid cells into semi-mature DCs and impairs T cell activation (150) (**Figure 2**). Moreover, the role of modulating differentiation by IL-6 was also examined in the breast cancer (148). Glioma-derived EVs down-regulate the expression of IL-12p70 in immortalized DCs, which orchestrates the maturation and differentiation of DCs (151).

## 4 Neutrophils

Neutrophils, abundant in circulation, are indispensable for an immune response due to their dual role of both affecting innate immunity and modulating adaptive immunity (152). The complicated function of neutrophils in the innate immune response includes forming neutrophil extracellular traps (NETs), polarization to a different state, phagocytosis, co-regulation with T cells, and so on. Interestingly, NETs are a double-sided sword in the immune response. On the one hand, they neutralize and ensnare microbiotics to against infection. On the other hand, they have adverse such as promoting thrombosis, tumor metastasis, and inflammation that causes organ and vascular damage (**Figure 2**). In addition, neutrophils also exert a dual effect on tumors by polarization to N1 or N2 phenotypes. In TME, stimulators including TGF-β and IFN-β respectively switch the phenotype of tumor-associated neutrophils (TANs) into N1 and N2 phenotypes (153) (**Figure 2**). The N1 phenotype shows an anti-tumor effect *via* enhancing apoptosis and secreting pro-inflammatory cytokines, while the N2 phenotype promotes tumor development and suppresses immune responses (154) (**Figure 2**).

### 4.1 TDEVs promote neutrophils NETs formation

TDEVs engulfed by neutrophils target NETs to promote thrombosis. A previous study has shown that tumor microparticles carrying tissue factor (TF) promoted cancer-associated deep vein thrombosis (DVT) initiation by adhering to NETs in a mouse model with pancreatic cancer (155). Ana C. Leal et al. showed that 4T1 murine breast tumor derived-EVs contribute to the prothrombotic state *via* inducing NETs formation by neutrophils stimulated by G-CSF in the murine breast cancer model (**Figure 2**). Moreover, there is a dose-dependent procoagulant property of 4T1 derived-EVs and this progress relies on the ability to recruit TDEVs by NETs (156). Exposure to TDEVs which bear gDNA induces TF activation in leukocytes, along with the upregulation of IL-8 (157). However, the function that TDEVs stimulated NETs promoting tumor growth is still under investigation.

### 4.2 TDEVs modulate neutrophils polarization

In addition to stimulating the formation of NETs, TDEVs play an important role in regulating the polarization of neutrophils. In CRC, TDEVs carrying oncogene circPACRGL promote the differentiation of neutrophils from N1 to N2 by regulating the miR-142-3p/miR-506-30-TGF-β1 axis (158). circRNA PACRGL swallowed by neutrophils binds to miR-142-30/miR-506-3p to inhibit the post-transcriptional control of mature mRNA and therefore TGF-b1 expression is downregulated, which induces neutrophils into the N2 phenotype (**Figure 2**). In addition, TDEVs induce neutrophil polarization to the N2 phenotype *via* the NF-κB pathway in gastric cancer and CRC (159, 160). Gastric cancer-cell derived EVs carrying high mobility group box-1 (HMGB1) bind with TLR or receptor for advanced glycation end products (RAGE) to activate NF-κB signaling, which induces the phosphorylation of downstream proteins including p65, STAT, and ERK and upregulates the expression of inflammatory factors such as IL-1β, IL-6, IL-8, OSM, and TNFα (**Figure 2**). Moreover, these pro-tumor effects of EVs can be blocked by NF-κB inhibitors (159). In HCC, TDEVs regulate the phenotype of neutrophils into N2, but the exact mechanism needs further investigation (161). Taken together, these data suggest that TDEVs induce neutrophils to polarize into the pro-tumor state of the N2 phenotype in various signaling pathways.

### 4.3 TDEVs regulate neutrophils’ other functions

Other functions of neutrophils can also be regulated by TDEVs. For example, the lifespan of neutrophils was prolonged by EVs from CRC stem cells by modulating the expression of IL-1β *via* the NF-κB signaling axis. EVs with tri-phosphate RNAs, acting as pathogen-associated molecular pattern (PAMP) molecules, interact with PRRs and activate the NF-κB pathway with elevated expression of nuclear p65 and IL-1β [20]. In addition, PD-L1 on neutrophils is upregulated by gastric cancer cell-derived EVs, activated by HMGB1 *via* phosphorylating STAT3 and downstream molecules, which suppresses T-cell immunity to have a pro-tumor influence (162) (**Figure 2**). More changes in neutrophil function by TDEVs need further investigation.

## 5 NK cells

NK cells are a subset of type 1 ILCs with surface markers CD3^-^ and CD19^-^, but are CD56^+^ and CD16^+^. They originate from common lymphoid progenitor (CLP) cells in the bone marrow and are widely distributed in the blood, peripheral lymphoid tissue, liver, spleen, and other organs, accounting for 5-10% of peripheral blood mononuclear cells (163, 164). Activating and inhibitory receptors are co-expressed on the surface of NK cells, which can bind to MHC I molecules expressed on the surface of one’s cells. NK cells also express NKG2D and natural cytotoxicity receptors (NCR) (NKp30, NKp44, and NKp46) (165). They are activating receptors that do not interact with MHC I. Cancerous cells decrease the expression of MHC I and cause a loss of inhibitory receptor function, referred to as the “missing-self” mode. Meanwhile, tumor cells overexpress ligands of NKG2D and NCR, providing sufficient targets for activating receptors *via* the “induced-self” mode (166, 167). NK cells are activated through the above two modes and kill tumor cells by releasing perforin, granzyme, TNF-α, or FasL (168). In addition, as a group of type 1 ILCs, NK cells synthesize and secret IFN-γ to play a role in the anti-infection and immune regulation (169, 170).

In most cases, TDEVs exert an influence on NK cells through NKG2D ligands (MICA, MICB, ULBP-1, ULBP-2, or ULBP-3) existing on the surface of EVs. NKG2DLs downregulate NKG2Ds floating on the surface of NK cells and block cell activation, resulting in the damage of NK cytotoxicity (171, 172) (**Figure 2**). Besides NKG2DLs, TGF-β1 (173, 174) and some other immunosuppressive proteins (PD-L1, CD39, CD73, FasL, LAP-TGFβ, TRAIL, CTLA-4) (175–177) are common cargoes of TDEVs, which act the same way as NKG2DLs do (**Figure 2**). TGF-β in EVs has another mechanism to inhibit the activation of NK cells: interacting with its receptors on the surface of NK, activating the TGFβ-Smad2/3 pathway, and promoting the phosphorylation of Smad2/3 (178, 179). Furthermore, soluble ligand BAG6/BAT3 was found to exist in chronic lymphocytic leukemia patients’ blood (180). Once BAG6/BAT3 is expressed on the surface of EVs it interacts with the activating receptor NKp30 of NK cells and induces cell stress through the nSmase2-dependent pathway to suppress NK cytotoxicity (180).

Some cytokines in TDEVs have a dual effect on NK cells. For example, genetically modified myeloid leukemia cell line K562 expresses IL-15, IL-18, and 4-1BBL (TNFSF9) on the surface of its EVs. These proteins stimulate NK cells to proliferate and enhance the cytotoxicity of NK cells within 4 hours. However, as time goes by (48 hours), the cytokines reduce NK cytotoxicity *via* the inhibition of activated receptors (NKG2D、NKp44) and the promotion of inhibitory receptors (NKG2A) (181). Moloudizargari et al. also reported the bifacial effect of myeloma derived-EVs on NK cells (181, 182).

The RNA component in TDEVs is also participating in regulating NK cells’ function. CRC-derived EVs containing lncRNA SNHG10 increase INHBC expression and then suppress the activation of NK (183). MiRNA-378a-3p in EVs is induced in tumors undergoing radiotherapy, leading to the reduction of granzyme-B secretion and loss of activity in NK cells (184). EVs from HCC cells deliver circUHRF and affect NK cells through three different ways to achieve immunosuppression and drug resistance. Firstly, decreasing IFN-γ and TNF-α secretion of NK cells. Secondly, degrading miR-449c-5p to foster the expression of TIM-3. Based on the fact that circRNAs usually work as miRNA sponges, studies indicated that circUHRF and miR-449c-5p may target each other in human NK-92 cells, thus impairing their function. Finally, there is a finding that the high levels of circUHRF1 in EVs can increase the connection with limited NK cell proportion and tumor infiltration (185).

## 6 Conclusions and perspectives

In recent years, EVs have become a popular topic in cancer and immunity research due to their complicated functions in regulating TME and their important role in mediating cell-to-cell communication (186, 187). Almost all cells including immune cells and cancer cells can generate EVs with common or specific cargoes which interact with recipient cells to further affect their functions and tumorigenesis (188, 189). In this review, we summarize the regulation of innate immune cells including macrophages, DCs, NK cells, and neutrophils by TDEVs (**Figures 1**, **2**). Overall, TDEVs exert a dual effect on immune responses and tumor development. For example, the secretion of pro-inflammation cytokines by macrophages and cytotoxicity of NK cells is either up-regulated or down-regulated by TDEVs. Moreover, TDEVs induce macrophage polarization to different states (functions as pro-tumor or anti-tumor), further influencing tumorigenesis, metastasis, and so on. Previous studies have demonstrated that the specific function of TDEVs mainly depends on their bioactive cargoes and tumor stage. For instance, at the beginning of tumorigenesis, EVs stimulate TAM and up-regulate cytokines that beneficial for angiogenesis and tumor metastasis. However, in the context of metastasis, anti-tumor responses such as cytotoxicity and phagocytosis are promoted by TDEVs. Based on these previous findings, the expression of innate immune cell markers and related molecules can be used as step-change indicators of diagnosis and prognosis in clinical treatment (190).

Various surface proteins and cargo components in TDEVs have been found to play an essential role in the regulation of macrophage polarization. Of the cargo components, most of the studies so far have been on miRNAs (**Table 1**). Other RNAs, proteins, and cytokines also target recipient cells to modulate their functions *via* direct binding with receptors or regulating corresponding gene expression. While the specific mechanism is still not completely known, NF-κB pathway and PTEN/PI3K/AKT pathways have been recognized as crucial signaling pathways in regulating immune responses. Therefore, key factor inhibitors can pharmacologically antagonize the effect of TDEVs. Moreover, there are also numerous studies concerning modulation of the formation, circulation, and absorption of TDEVs in anticancer therapies (191).

Although existing studies have provided many insights about the regulation of innate immunity by TDEVs, there are still many limitations. Firstly, the studies investigating TDEVs’ modulation of the function of innate immune cells like mast cells, eosinophils, and basophils remain inadequate. Secondly, more detailed mechanisms of TDEVs functions on innate immunity are still under investigation, and the results of these studies will be vital for identifying new cancer targets. Additionally, the majority of current studies are *in vitro* experiments, and more *in vivo* investigations are supposed to be conducted to better demonstrate the effects of TDEVs in cancer progression (192). TDEVs mediating intercellular communication between cancer and innate immune cells is a promising field, which can shed a spotlight on novel cancer therapies. We hope more investigations will be conducted to promote this progression.

## Author contributions

ZL conceived and designed this review. SW and JS draft the manuscript. SW, JS, RD, and ZL revised and edited the manuscript. All authors approved the final version of the manuscript. All authors contributed to the article and approved the submitted version.

## Funding

This work was supported by the Research Start-up Fund of the Seventh Affiliated Hospital, Sun Yat-sen University (ZSQYBRJH0021 to ZL); Fundamental and Applied Basic Research Fund of Guangdong Province (2021A1515110512 to ZL); General project of Shenzhen Science and Technology Innovation Committee (JCYJ20210324134612035 to ZL); Fundamental Research Funds for the Central Universities, Sun Yat-sen University (22qntd3703 to ZL).

## Conflict of interest

The authors declare that the research was conducted in the absence of any commercial or financial relationships that could be construed as a potential conflict of interest.

## Publisher’s note

All claims expressed in this article are solely those of the authors and do not necessarily represent those of their affiliated organizations, or those of the publisher, the editors and the reviewers. Any product that may be evaluated in this article, or claim that may be made by its manufacturer, is not guaranteed or endorsed by the publisher.

## Glossary

**Table:**

AMP	adenosine monophosphate
ADCC	antibody-dependent cell-mediated cytotoxicity
APC	antigen-presenting cells
APM	antigen-processing machinery
CRC	colorectal cancer
CDPs	common dendritic cell progenitors
CLP cells	common lymphoid progenitor cells
cDCs	conventional DCs
CTL	cytotoxic T lymphocyte
DVT	deep vein thrombosis
DCs	dendritic cells
ESCRT	endosomal sorting complex required for transport
EGFR	epidermal growing factor receptor
EMT	epithelial–mesenchymal transition
ECM	extracellular matrix
EVIR	extracellular vesicle-internalizing receptor
EVs	extracellular vesicles
FGF	fibroblast growth factor
HCC	hepatocellular carcinoma
HMGB1	high mobility group box-1
HNSCC	head and neck squamous cell carcinoma
ILCs	innate lymphoid cells
ILLs	innate-like lymphocytes
ISEV	International Society for Extracellular Vesicles
lEVs	large EVs
KDM6B	lysine demethylase 6B
LLC	Lewis lung cancer
MIF	macrophage migration inhibitory factor
MMP	matrix metalloproteinase
mEVs	medium EVs
MCP-1	monocyte chemoattractant protein 1
moDCs	monocyte-derived inflammatory DCs
MVBs	multi-vesicular bodies
NCR	natural cytotoxicityreceptor
NK	natural killer
NE	neutrophil elastase
NETs	neutrophil extracellular traps
NSCLC	non-small cell lung cancer
OSCC	oral squamous cell carcinoma
PDAC	pancreatic ductal adenocarcinoma
PAMP	pathogen-associated molecular pattern
PRRs	pattern recognition receptors
pDCs	plasmacytoid DCs
PDGF	platelet-derived growth factor
PGE2	prostaglandin E2
PTPRO	protein tyrosine phosphatase receptor type O
ROS/RNS	reactive oxygen/nitrogen species
RAGE	receptor for advanced glycation end products
RFXAP	regulatory factor X-associated protein
SIPA1	signal-induced proliferation-associated 1
sEVs	small EVs
TIM-3	T cell immunoglobulin and mucin domain 3
TIM-4	T cell immunoglobulin and mucin domain-4
TLR	Toll - like receptor
TA	tumor antigen
TME	tumor microenvironment
TAM	tumor-associated macrophages
TANs	tumorassociated neutrophils
TDEVs	tumor-derived EVs
TIDC	tumorinfiltrating dendritic cells
VEGF	vascular endothelial growth factor
